# Adaptive plasticity in activity modes and food web stability

**DOI:** 10.1371/journal.pone.0267444

**Published:** 2022-04-21

**Authors:** Akihiko Mougi

**Affiliations:** Institute of Agricultural and Life Sciences, Academic Assembly, Shimane University, Matsue, Japan; National Cheng Kung University, TAIWAN

## Abstract

Natural ecosystems are comprised of diverse species and their interspecific interactions, in contrast to an ecological theory that predicts the instability of large ecological communities. This apparent gap has led ecologists to explore the mechanisms that allow complex communities to stabilize, even via environmental changes. A standard approach to tackling this complexity-stability problem is starting with a description of the ecological network of species and their interaction links, exemplified by a food web. This traditional description is based on the view that each species is in an active state; that is, each species constantly forages and reproduces. However, in nature, species’ activities can virtually stop when hiding, resting, and diapausing or hibernating, resulting in overlooking another situation where they are inactive. Here I theoretically demonstrate that adaptive phenotypic change in active and inactive modes may be the key to understanding food web dynamics. Accurately switching activity modes can greatly stabilize otherwise unstable communities in which coexistence is impossible, further maintaining strong stabilization, even in a large complex community. I hypothesize that adaptive plastic change in activity modes may play a key role in maintaining ecological communities.

## Introduction

May [1] mathematically demonstrated that large ecosystems are comprised of diverse species and their interactions become inherently unstable and fragile in response to environmental fluctuations, unlike large natural ecosystems. This contradiction between nature and theory, labeled as the complexity–stability debate, has been investigated by ecologists in an attempt to understand the maintenance of ecological communities in nature [2–5]. A major approach to tackling this problem is to reveal the relationship between the ecological network exemplified by a food web (a classical representation of “who eats whom” in the community), and the stability of population dynamics [6,7]. Based on this traditional approach, for half a century, ecologists have proposed diverse mechanisms that allow large ecological communities to remain stable [8–15]. However, it remains unclear whether this ecological view is applicable to real nature, since along this line, each species within a community always actively forages and reproduces, omitting another world in which each species is not active and has almost no species interactions.

In nature, organisms should have both active and inactive modes [16–18]. Animals actively search for resources, prey, mates, and opportunities to reproduce. Active modes are expected to contribute to the population growth, but they can also increase the opportunity to encounter predators, which negatively affect population growth. Thus, active modes are expected to play a role in driving population fluctuations. In this context, the active mode has been a basal model traditionally used to describe the population dynamics in ecological communities. There is no doubt that the standard community dynamics model, due to its analytical simplicity, has greatly contributed to our understanding of how ecological communities are maintained [5]. On the other hand, it would be impossible for organisms to always be active. Organisms spend significant time in inactive modes for diverse reasons, such as resting, recovering from injury, hiding from enemies, diapausing, and hibernating [16]. The inactive mode largely affects community dynamics in several ways. Once the animal is not active, (i) the interaction strengths will be weakened or even vanish. Because the animal is in safety mode, predation risk from enemies becomes low, but the animal loses the opportunity to forage; and (ii) loses the chance to mate so reproduction will decrease, which will decrease the population growth rate. Generally, this is a cost of inactive modes. For example, if the inactive mode is due to a morphological-inducible defense, the maintenance cost of the defensive phenotype can decrease the growth rate [19–21]. Thus, the inactive mode itself is almost never expected to contribute to population dynamics. More importantly, the expected effect of the inactive mode is related to the interaction network. Once a species switches from active to inactive mode, (iii) the node (focal species) within the interaction network will disappear from the active world, resulting in a disappearance of interaction links; this implies a decrease in community complexity, which can largely affect community stability [1].

**Fig 1:**
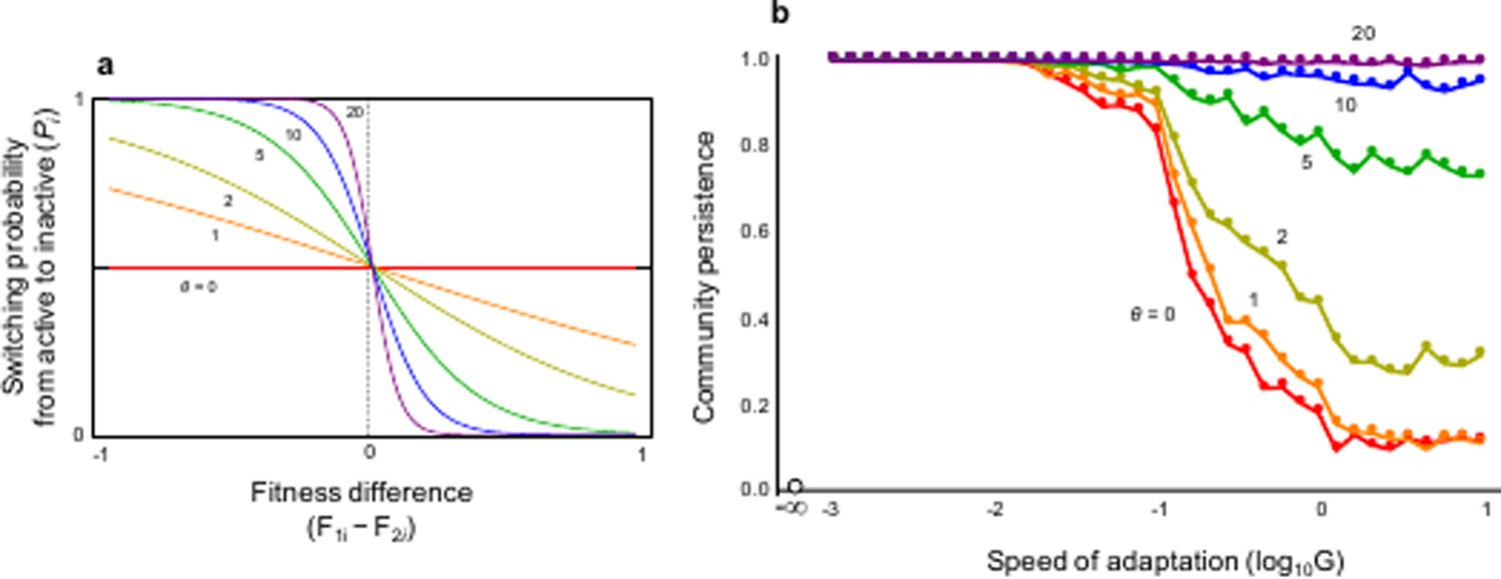
Plastic changes in activity modes and community stability. (a) Switching function of activity modes, *P*_*i*_ (Eq 2 in Methods); (b) Effects of the speed and accuracy of phenotypic switching on community stability. See S8 Fig for the confidence interval. The white circle in (b) is the result of no adaptation. N = 40 and C = 0.3. Parameters are *R =* 0.04, *E* = 0.01, *A*_1_ = 0.1, and *A*_2_ = 0.05.

Here, using a food web model, I demonstrate that switching activity modes in organisms greatly affects community dynamics stability and plays a key role in maintaining complex communities. The model proposed in this study is based on a food web comprising *N* species, any pair of which is connected with the probability *C* (connectance). Population dynamics (population sizes of active and inactive modes in each species are respectively represented by *X*_1*i*_ and *X*_2*i*_, where *i* = 1,…,*N*) are driven by interspecific prey-predator interactions (see Materials and methods). A cascade food web was assumed [22] (a random food web was also tested). Two activity modes were considered: active and inactive. Population growth rates, reproduction rates, and interaction strengths in the inactive mode are lower than in the active one. Each species can switch between activity modes, based on the difference between the fitness (or per capita growth rate) of each activity mode [23,24]. A degree of adaptation is characterized by the speed and accuracy of switching between activity modes. The parameter *G* controls the speed of plastic changes in activity modes. *θ* denotes the plastic sensitivity to differences in fitness between activity modes (accuracy), and may be interpreted as the ability to catch environmental information; a larger *θ* represents a higher certainty of environmental information and *θ*  =  0 indicates that no environmental information is available. Hence, when *θ* = 0, phenotypic changes are random and when *θ* is high, the model approaches a step function of differences in each fitness (Fig 1A). I controlled the adaptation level (*G* and *θ*) to examine how activity mode affects the stability of ecological communities as evaluated by community persistence, with the probability that all species persist for a given time [24,25] (see Materials and methods for further details).

## Materials and methods

A cascade food web [22] is considered, in which pairs of species *i* and *j* (*i*, *j* = 1,…, *N*) are connected by a trophic interaction with a probability of *C*, which is defined as the proportion of realized interaction links *L* in the possible maximum interaction links *L*_*max*_ of a given network model (*L = CL*_*max*_). For each pair of species, *i*, *j* = 1,…, *N* with *i* < *j*, species *i* never consumes species *j*, whereas species *j* may consume species *i*. To examine the generalization of the main result, random food webs were also tested (S3 Fig). The maximum link number *L*_max_ is calculated from *N*(*N*−1)/2 in both the random and cascade models. The food web model is defined by the following ordinary differential equation:

$$dX_{1i}/dt=r_{1i}X_{1i}-s_{i}X_{1i}{}^{2}+\Sigma_{j}M_{1ij}X_{1j}X_{1i}+G(-\gamma_{i}P_{i}X_{1i}+\gamma_{i}(1-P_{i})X_{2i}),$$ (1A)

$$dX_{2i}/dt=r_{2i}X_{2i}-s_{i}X_{2i}{}^{2}+\Sigma_{j}M_{2ij}X_{2j}X_{2i}+G(-\gamma_{i}(1-P_{i})X_{2i}+\gamma_{i}P_{i}X_{1i}),$$ (1B)

where *X*_*ki*_ is the abundance of species *i*, *r*_*ki*_ is the intrinsic rate of change in species *i*, *s*_*i*_ is the density-dependent self-regulation of species *i*, and *M*_*kij*_ is the interaction coefficient between species *i* and *j*. Interaction coefficients are defined as *M*_*kij*_ = *e*_*kij*_*a*_*kij*_ and *M*_*kji*_ = −*a*_*kij*_, where *a*_*ij*_ is the consumption rate of resource species *j* by species *i* and *e*_*kij*_ (<1) is the conversion efficiency. The subscript *k* represents the activity modes, where 1 is active and 2 is inactive. The last two terms in r.h.s. of Eq (1) represent the dynamics due to plastic changes between the active and inactive modes. *G* is the constant parameter that controls the speed of plastic changes in activity modes, *γ*_*i*_ is the species-specific phenotypic change rate, and *P*_*i*_ is the switching probability from an active to inactive mode, described by the following non-linear function:

$$P_{i}=1/(1+Exp[\theta (F_{1i}-F_{2i})]),$$ (2)

where *F*_*ki*_ (*k =* 1 or 2) is the fitness of the active and inactive modes, defined as *F*_*ki*_ = *r*_*ki*_–*s*_*i*_*X*_*ki*_ + Σ_*j*_*M*_k*ij*_*X*_k*j*_. *θ* denotes the plastic sensitivity to differences in fitness between the activity modes, and may be interpreted as the ability to catch the information on environments. A larger *θ* represents a higher certainty of environmental information, while *θ*  =   0 indicates that no environmental information is available. Hence, when *θ* = 0, phenotypic changes are random and when *θ* increases, the model approaches a step function of differences in each fitness.

The active and inactive models are defined as follows: (i) *r*_1*i*_ *> r*_2*i*_ (= *Rr*_1*i*_, where *R* < 1 is a constant parameter that controls the degree of growth rate reduction from the active to inactive modes); this reflects a cost, due to the phenotypic change to the inactive modes and/or a reduction of the utilization of extra resources. (ii) *e*_1*ij*_ *> e*_2*ij*_ (= *Ee*_1*ij*_, where *E* < 1 is a constant parameter that controls the degree of conversion efficiency reduction from the active to inactive modes); this also reflects a cost due to the phenotypic change to the inactive modes. (iii) *a*_1*ij*_ *> a*_2*ij*_ (= *A*_2_*a*_1*ij*_, where *A*_2_ < 1 is a constant parameter that controls the degree of reduction of interaction strengths from active to inactive modes); this reflects that phenotypic change to the inactive modes makes each species more defensive, but reduces foraging activity. This considers a special case where the refuge in inactive mode is common among each species and very safety against active predators. If they use different habitats in active and inactive individuals, the interaction between active and inactive individuals would be rare. This strong assumption will be relaxed afterward.

It is assumed that *r*_1*i*_ = c_*i*_*r*, *a*_1*ij*_ = c_*i*_*A*_1_, and *γ*_*i*_ = c_*i*_, where c_*i*_ is a constant randomly determined from a uniform distribution (0.0–1.0), *r* is the absolute growth rate of the active mode (assumed as *r =* 5), and *A*_1_ is the absolute interaction strength (consumption rate) of the active mode. In this study, for convenience purposes, *A*_1_ and *A*_2_ are referred to as the interaction strength of the active and inactive modes, respectively.

In each iterated simulation, the initial species abundance and parameters were randomly selected from a uniform distribution (*X*_*ki*_, 0 to 1.0; c_*i*_, 0 to 1.0). The values for *s*_*i*_ and *e*_1*ij*_ were set to constants of 1 (rescaling the species abundances) and 0.2 [26,27], respectively. A positive growth rate in the absence of interaction links was used in all species to avoid a confounding effect, in which an increase in interspecific links decreases the number of heterotrophic species with no potential diet present in the web [14]. From a biological perspective, each species is either autotrophic or makes use of external resources.

Community persistence [24,25] was calculated by measuring the fraction of simulations in which all coexistent species (Σ_*k*_*X*_*ki*_ > 10^−13^ for all *i*) after a sufficiently long time (t = 5 × 10^3^, which corresponded to the time taken for community persistence to reach an asymptote) in 500 runs.

## Results and discussion

Consider an extreme case without inactive mode (*G* = 0 and *X*_2*i*_ = 0 for all *i*). Then, in a complex community with diverse species, virtually no community persists (white circle in Fig 1B), as shown via previous food web models. In a world where each species is always active, the population dynamics are predicted to be unstable.

Another case with the inactive mode (i.e., defense, but less foraging/reproduction) is considered. Here, an extreme case, where the inactive mode does not interact with active other species, is assumed first. In this situation, it is expected that inactive individuals remain completely at rest in a safe refuge. Once each species can change their activity mode (*G* > 0) or switch between active and inactive modes, the community can persist. The stabilization effects critically depend on the fitness sensitivity *θ* and adaptation speed *G*. Under slow adaptation (*G*≪1), the community becomes highly stable and virtually all communities persist, regardless of fitness sensitivity (Fig 1B). However, further increases in the speed of adaptation can alter the stabilization effect, depending on fitness sensitivity. Without sensitivity (*θ* = 0), stability decreases as the speed of adaptation increases (Fig 1B); the destabilization is so strong that almost no community can persist. However, if each species has high fitness sensitivity, the destabilization due to faster adaptation can vanish (Fig 1B). Under much faster adaptation, the times required for each species to overcome the less adaptive situation are short; thus, if they cannot accurately change their activity modes depending on the environmental situation, phenotypic changes would not play a stabilizing role in community dynamics. As such, under what conditions, in an alternate world, can adaptive phenotypic plasticity play a key role in stabilizing community dynamics?

**Fig 2:**
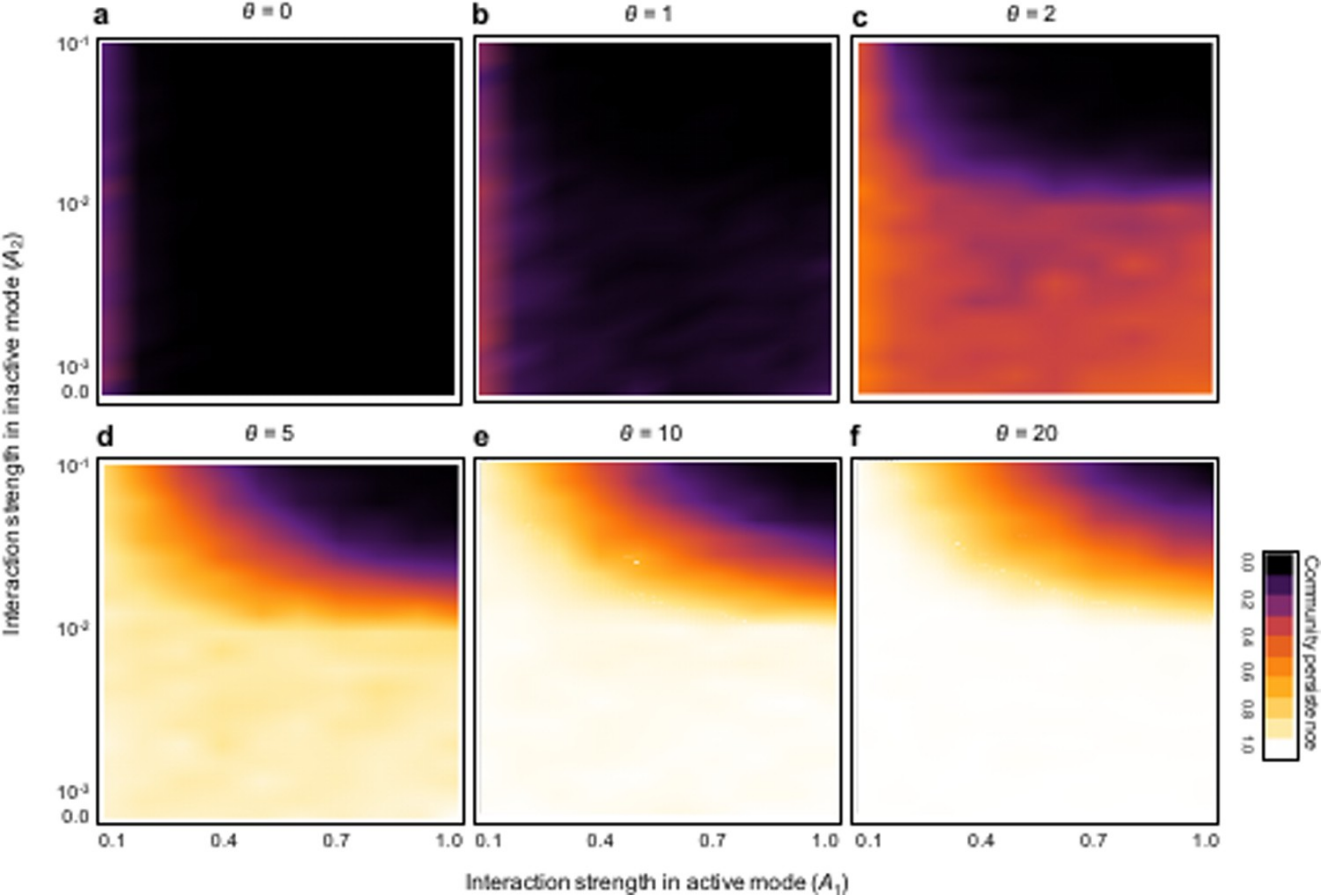
Effects of the interaction strengths of each activity mode on community stability, with the variation of switching accuracy (*θ*). Color represents the level of community stability as shown within panel (f). N = 40 and C = 0.3. Parameters are *R =* 0.04, *E* = 0.01, and *G* = 1.

As predicted by earlier studies [9,13,14], interaction strengths play a major role in community stability. Fig 2 illustrates the effect of the relationship of the interaction between active and inactive modes on community stability. When the interaction strengths in inactive modes are much lower than those in active modes, communities are likely to persist; the requirement of lower interaction strengths in inactive modes to ensure high stability weakens as the interaction strengths in active modes decrease or fitness sensitivity increases. In addition, with high fitness sensitivity, high community stability is maintained in the broad parameter space of interaction strengths (Fig 2).

In the above analysis, I have assumed lower growth or reproduction in inactive modes than in active modes, in addition to lower interaction strengths in inactive modes. Here, I relax the strong conditions. The analysis demonstrates that stability is almost not influenced by the asymmetry of growth and reproduction rates between the two modes (S1 Fig). This suggests that stabilization due to adaptive phenotypic plasticity critically depends on the asymmetry of interaction strengths between the two phenotypic modes.

**Fig 3:**
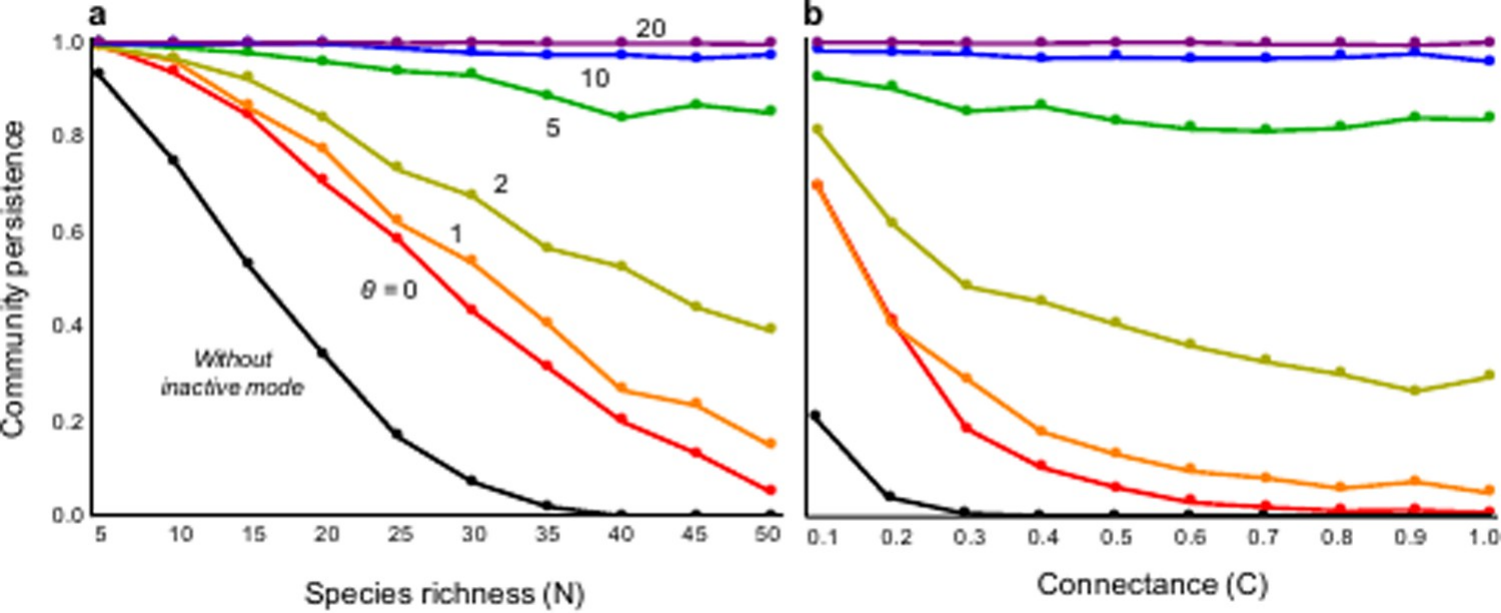
Relationships between food web complexity and stability. (a) Effects of species richness (N), C = 0.3; (b) Effects of connectance (C), N = 40. See S8 Fig. for the confidence interval. Black lines represent cases with no inactive mode. Color represents different values of *θ*. Parameters are *R =* 0.04, *E* = 0.01, *A*_1_ = 0.1, *A*_2_ = 0.01, and *G* = 1.

Adaptive phenotypic plasticity has an intriguing consequence on the ongoing complexity-stability debate. Increased complexity (high species richness and connectance) destabilizes community dynamics and can cause species extinctions in food webs without inactive modes (Fig 3). However, with inactive modes, phenotypic change weakens or even cancels the destabilization effects of complexity. Particularly with high sensitivity, communities are not at all destabilized by increased complexity, and can maintain high community stability (Figs 3 and S2). This insensitivity in response to an increase in complexity can be also observed in other network types, like the random model (S3 Fig).

Here, the model is extended into more general situations. The above analysis assumed no interactions between the active and inactive modes. In addition, intraspecific interaction (self-regulation) does not change among the different activity modes. These strong assumptions are relaxed, and it is first assumed that intraspecific interaction becomes weak in the inactive mode. Although intraspecific competition among the inactive modes should not weaken if species compete for a refuge, it should do so if the inactive mode is a defensive phenotype, such as inducible defense, because species do not use the refuge and activity is low. Second, the following relations between consumption rates are assumed: *a*_11*ij*_ *> a*_12*ij*_, *a*_21*ij*_ and *a*_12*ij*_ *> a*_21*ij*_, *a*_22*ij*_, where *a*_*klij*_ is the consumption rate of active prey *j* by active species *i* if *k = l* = 1, that of inactive prey *j* by active species *i* if *k =* 1 and *l* = 2, that of active prey *j* by inactive species *i* if *k =* 2 and *l* = 1, and that of inactive prey *j* by inactive species *i* if *k = l* = 2, respectively. This is because active individuals encounter other individuals more frequently. In particular, active predators are likely to find active prey more frequently than inactive prey, while inactive predators are less likely to find inactive prey than active prey. The abovementioned novel assumptions corroborate with the main results.

**Fig 4:**
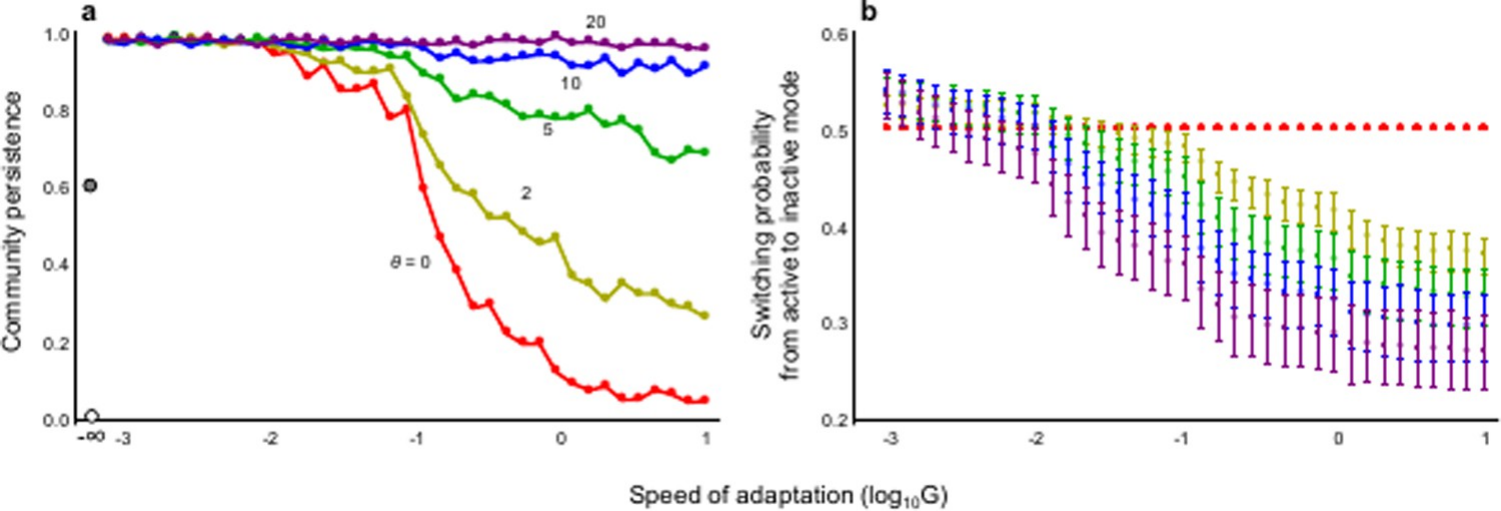
Plastic changes in activity modes and community stability in the case of interaction between the active and inactive modes. (a) Effects of the speed and accuracy of phenotypic switching on community stability; (b) Effects of the speed and accuracy of phenotypic switching on mean switching probability. See S8 Fig for the confidence interval in (a). The white circle in (a) is the result of no adaptation and the gray circle in (a) is the result of a system with only inactive mode. The mean switching probability is calculated as the mean of species mean *P*_*i*_ in a community after a sufficiently long time (at an equilibrium) in simulation runs. Each bar in (b) represents the standard deviation. N = 40 and C = 0.3. Parameters are *A*_12_ = 0.01, *A*_21_ = 0.005 and *A*_22_ = 0.001. Other parameters are the same as those in S4 Fig.

The result shows that a key species interaction for community stability is that between active predator and inactive prey. As the interaction strength of an active predator to inactive prey decreases, the stability increases (S4 Fig). However, the other interactions (inactive predator to active prey and inactive predator to inactive prey) have less or virtually no effects on stability. This suggests that the adaptive plasticity in activity modes can contribute to community stability if the inactive mode strongly acts as a defense. In fact, in such a case, the main results are held. High adaptation ability (high *θ* and *G*) is likely to stabilize the system (Fig 4A). Even if *θ* varies, its effect on stability does not change qualitatively (S5 Fig). In addition, complex communities with rich species and dense interactions can maintain high community stability due to adaptive plasticity (S6 Fig).

In order to understand how the phenotypic changes in the activity modes contribute to community stability, the analysis of switching dynamics among the activity modes is particularly useful. Phenotypic switching is largely affected by adaptive speed and accuracy (S7 Fig). When adaptive speed is slow, the switching probability from the active to inactive mode is nearly 0.5 at the equilibrium, irrespective of accuracy (Fig 4B). Due to a long stay in the active mode, each species should face a high predation risk. In such a situation, switching to the inactive mode is likely to be adaptive. Because inactive modes are more stable (gray circle in Fig 4A), the inactive mode acts to rescue the persistence of the whole system. In contrast, when adaptive speed is fast, the equilibrium switching probability from the active to inactive mode decreases, and largely differs depending on the accuracy (Fig 4B). As accuracy increases, the switching to the inactive mode decreases. Due to a short stay in each mode, individuals are not likely to receive a merit of each activity mode if they inaccurately switch between the two modes. If the accuracy is high, inactive individuals would be able to avoid a high predation risk and active individuals would be able to enjoy high foraging rate and growth rate by avoiding a long-term predation risk, resulting in the low switching to the inactive mode. In this situation, interaction strengths in the active modes become weak because the species interacting in the active mode may not be present. On the other hand, a high switching from the inactive to active mode will act to rescue the persistence of the whole system.

The present study demonstrated that adaptive plastic change in activity modes can play a key role in stabilizing complex food webs, with weak interactions in inactive mode and quick and accurate changes in activity modes being important stabilizing factors. The adaptive plasticity causes much higher stability than the system with only inactive individuals (Fig 4A). The stabilization effect of adaptive plasticity in activity is so strong that inherent instability due to community complexity can vanish, providing a new mechanism of community maintenance. Roughly speaking, the present framework can relate to diverse temporal and spatial phenomena in organisms, including behavioral refuge use, inducible morphological defense, resting, diapausing, and hibernating, suggesting a general mechanism for maintaining ecological communities.

The present theory is intimately linked to earlier works on adaptive behavior and its consequences on ecological community dynamics [28]. Previous studies have predicted that adaptation in foraging [14,29,30] and defense [31–33] commonly has a stabilizing effect on community dynamics. With adaptive foraging, adaptive consumers decrease their foraging efforts against rare resources, allowing for the recovery of rare species. In a similar way, if defense efforts are specifically allocated to each predator, adaptive defense enables the recovery of rare consumers whose resources decrease their avoidance efforts against them. These can be seen as apparent mutualism phenomena that emerge due to the inherent frequency dependence of adaptive trophic behavior [28]. Adaptive behaviors can increase their own and other species’ fitness, resulting in a whole system balance. In the present model, via different ways due to effort allocations, a similar stabilization would occur. If a focal species largely decreases resources, it is expected to suppress its own activity to wait for resource recovery. On the other hand, if a focal species is overly attacked by predators, it is expected to seek refuge until predation rates decrease. The former and latter situations would indirectly enable the recovery of rare resources and rare consumers, respectively. In this sense, the adaptive plasticity of activity is a comprehensive mechanism of adaptive behaviors. Furthermore, it has a more important stabilization mechanism, in contrast with earlier theories. Activity changes can temporally decrease species node and interaction links within an active community network; switching from active to inactive modes implies a disappearance of the species node and the subsequent disappearance of the related interaction links. Hence, in a complex system with many species and interaction links, inactivity can largely decrease system complexity, even temporarily, with the nodes and links repeatedly appearing and disappearing like flashing fireflies. The key point here is that the system becomes simple, and a simple system is more stable than a complex one, as predicted by May [1]. This is more critical in more complex systems, as they are inherently more unstable than simpler systems [1]. In other words, the present theory supports May’s prediction.

The present result has an important implication for the role of adaptive defense in community dynamics. The “refuge” has received high attention in earlier ecology works, especially in relation to the stabilization of predator-prey interactions [34]. However, it remains unclear whether stabilization works in more complex systems. In this sense, the present study links the classic, important concept of population ecology to community ecology. Inducible defense [35], phenotypic changes activated by a previous encounter with an enemy that confer some resistance to subsequent attacks, is a type of refuge. The plastic activity changes in the present model can also be interpreted as inducible defense. Inactive modes may be considered as a morphological or behavioral defensive phenotype. Previous studies have shown that inducible defense can greatly stabilize predator-prey dynamics [36–38]. Given the present results, strong stabilization may also work in complex systems. On the other hand, few studies have examined adaptive defense in complex food webs [31–33], some of which have shown that adaptive defense effort allocations do not have much of a stabilization effect on complex systems, compared with adaptive foraging. As discussed above, increased defense allocation to offensive consumers can contribute to community stability due to apparent mutualism; however, increased consumers can potentially increase the likelihood that resource competition between consumers overcomes apparent mutualism, resulting in the destabilization of complex systems, contrary to the present theoretical prediction. Speed and accuracy of the adaptive phenotypic changes are critical for community stability. The development of further mechanistic modeling considering condition-dependent adaptive changes in these two parameters [39,40] represents a future challenge. Nevertheless, the simple model presented in this study may have important implications for biological conservation. Evolutionary traps caused by anthropogenically-driven environmental changes, which cause maladaptive fitness between environments [41], may destabilize ecological communities.

## Supporting information

S1 Fig Effects of growth rates and reproduction rates on community stability. *R* and *E* are changed. Colors represent different values of *E* (red: 0.01, orange: 0.2, yellow; 0.4, green: 0.6, blue: 0.8, purple: 1.0). Other parameters are identical to those in Fig 1B.
(TIFF)

S2 Fig Relationships between food web complexity and stability with varying adaptation speed and accuracy. Note that in (a) and (f), *θ* did not affect the results (it was always stable). Parameters are identical to those in Fig 3.
(TIFF)

S3 Fig Relationships between food web complexity and stability in the random food web model. (a) Effects of species richness (N). I assume C = 0.3. (b) Effects of connectance (C). I assume N = 40. Black lines represent cases without inactive modes. Color represents deferent values of *θ*. Parameters are *R =* 0.04, *E* = 0.01, *A*_1_ = 0.1, *A*_2_ = 0.01, and *G* = 1.
(TIFF)

S4 Fig Effects of interactions among active and inactive modes on stability. (a) *A*_12_ = 0.01. (b) *A*_12_ = 0.02. (c) *A*_12_ = 0.05. (d) *A*_12_ = 0.1. *A*_*kl*_ is redefined as: *a*_11*ij*_ = c_*i*_*A*_11_, *a*_12*ij*_ = *A*_12_*a*_11*ij*_, *a*_21*ij*_ = *A*_21_*a*_11*ij*_, and *a*_22*ij*_ = *A*_22_*a*_11*ij*_, where *A*_11_ is the absolute interaction strength (consumption rate) of the active predator to active prey (a normal interaction strength), and *A*_12_, *A*_21_ and *A*_22_ are constant parameters that controls the degree of reduction of interaction strengths from normal to the other three cases. Different colors represent different values of *A*_21_. In (a), blue, red, yellow, and green are *A*_21_ = 0.001, 0.002, 0.005, and 0.01, respectively; in (b), blue, red, yellow, and green are *A*_21_ = 0.001, 0.002, 0.005, and 0.02, respectively; in (c), blue, red, yellow, and green are *A*_21_ = 0.001, 0.002, 0.01, and 0.05, respectively; and in (d), blue, red, yellow, and green are *A*_21_ = 0.001, 0.002, 0.01, and 0.1, respectively. N = 40 and C = 0.3. Parameters are *s*_1_ = 1, *s*_2_ = 0.5, *A*_11_ = 0.1, *R =* 0.04, *E* = 0.01, *θ* = 20, and *G =* 1.
(TIFF)

S5 Fig Effects of variation of *θ* on community stability. *θ*_*i*_ values are randomly chosen by a uniform distribution with a mean shown on the horizontal axis. Parameters are the same as those in Fig 4.
(TIFF)

S6 Fig Relationships between food web complexity and stability in a system with the interactions between active and inactive modes. (a) Effects of species richness (N), C = 0.3; (b) Effects of connectance (C), N = 40. Parameters are *s*_1_ = 1, *s*_2_ = 0.5, *A*_11_ = 0.1, *A*_12_ = 0.01, *A*_21_ = 0.005, *A*_22_ = 0.001. *r =* 1, *R =* 0.2, *E* = 0.01, and *G =* 1.
(TIFF)

S7 Fig Transient dynamics of switching probability *P*_*i*_. (a) *θ =* 5, *G =* 0.001; (b) *θ =* 5, *G =* 10; (c) *θ =* 20, *G =* 0.001; (d) *θ =* 20, *G =* 10. Each color represents a species. Other parameters are the same as those in Fig 4.
(TIFF)

S8 Fig Confidence interval of the main results. (a), (b), (c) and (d) correspond to Fig 1B, Fig 4A, Fig 3A and Fig 3B, respectively. Bar indicates the confidence interval.
(TIFF)

S1 File (DOCX)
