# Supplementary figures and images for: Adaptive plasticity in activity modes and food web stability
Source: PLoS One. 2022 Apr 21;17(4):e0267444. doi: 10.1371/journal.pone.0267444 (PMC9022794; doi:10.1371/journal.pone.0267444)

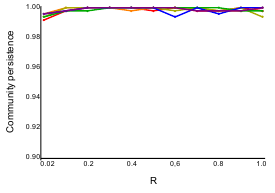

Supplement: S1 Fig — R and E are changed. Colors represent different values of E (red: 0.01, orange: 0.2, yellow; 0.4, green: 0.6, blue: 0.8, purple: 1.0). Other parameters are identical to those in Fig 1B. (TIFF) [file pone.0267444.s001.tiff]

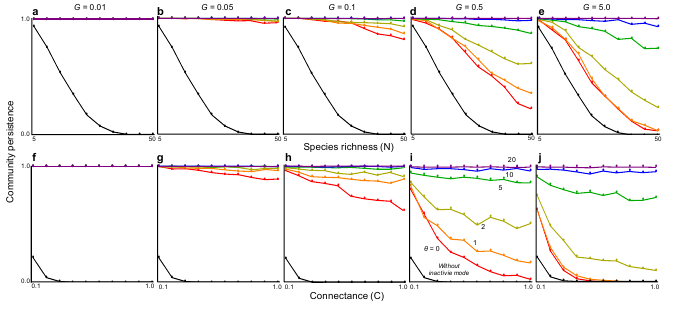

Supplement: S2 Fig — Note that in (a) and (f), θ did not affect the results (it was always stable). Parameters are identical to those in Fig 3. (TIFF) [file pone.0267444.s002.tiff]

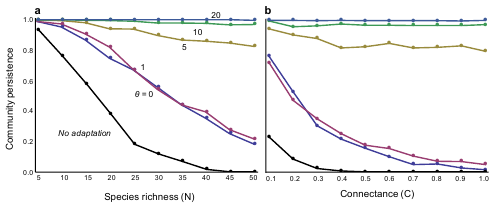

Supplement: S3 Fig — (a) Effects of species richness (N). I assume C = 0.3. (b) Effects of connectance (C). I assume N = 40. Black lines represent cases without inactive modes. Color represents deferent values of θ. Parameters are R = 0.04, E = 0.01, A1 = 0.1, A2 = 0.01, and G = 1. (TIFF) [file pone.0267444.s003.tiff]

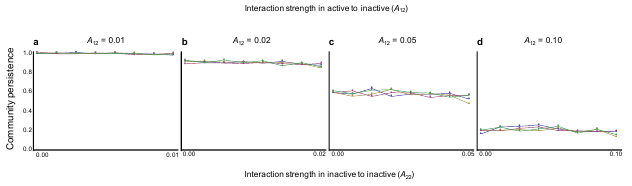

Supplement: S4 Fig — (a) A12 = 0.01. (b) A12 = 0.02. (c) A12 = 0.05. (d) A12 = 0.1. Akl is redefined as: a11ij = ciA11, a12ij = A12a11ij, a21ij = A21a11ij, and a22ij = A22a11ij, where A11 is the absolute interaction strength (consumption rate) of the active predator to active prey (a normal interaction strength), and A12, A21 and A22 are constant parameters that controls the degree of reduction of interaction strengths from normal to the other three cases. Different colors represent different values of A21. In (a), blue, red, yellow, and green are A21 = 0.001, 0.002, 0.005, and 0.01, respectively; in (b), blue, red, yellow, and green are A21 = 0.001, 0.002, 0.005, and 0.02, respectively; in (c), blue, red, yellow, and green are A21 = 0.001, 0.002, 0.01, and 0.05, respectively; and in (d), blue, red, yellow, and green are A21 = 0.001, 0.002, 0.01, and 0.1, respectively. N = 40 and C = 0.3. Parameters are s1 = 1, s2 = 0.5, A11 = 0.1, R = 0.04, E = 0.01, θ = 20, and G = 1. (TIFF) [file pone.0267444.s004.tiff]

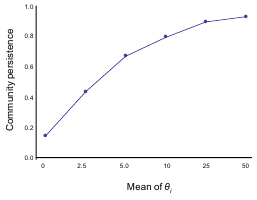

Supplement: S5 Fig — θi values are randomly chosen by a uniform distribution with a mean shown on the horizontal axis. Parameters are the same as those in Fig 4. (TIFF) [file pone.0267444.s005.tiff]

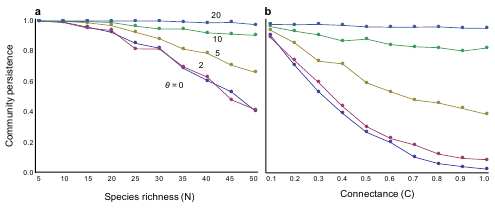

Supplement: S6 Fig — (a) Effects of species richness (N), C = 0.3; (b) Effects of connectance (C), N = 40. Parameters are s1 = 1, s2 = 0.5, A11 = 0.1, A12 = 0.01, A21 = 0.005, A22 = 0.001. r = 1, R = 0.2, E = 0.01, and G = 1. (TIFF) [file pone.0267444.s006.tiff]

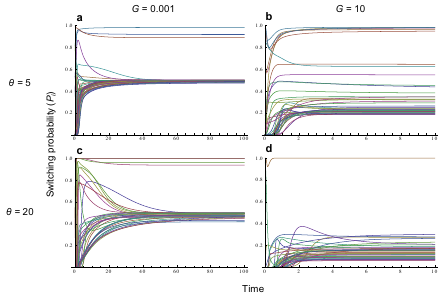

Supplement: S7 Fig — (a) θ = 5, G = 0.001; (b) θ = 5, G = 10; (c) θ = 20, G = 0.001; (d) θ = 20, G = 10. Each color represents a species. Other parameters are the same as those in Fig 4. (TIFF) [file pone.0267444.s007.tiff]

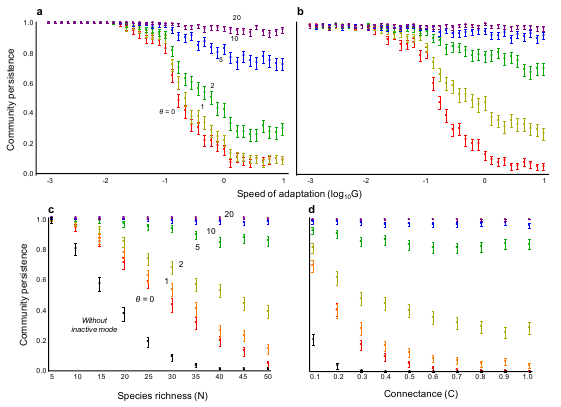

Supplement: S8 Fig — (a), (b), (c) and (d) correspond to Fig 1B, Fig 4A, Fig 3A and Fig 3B, respectively. Bar indicates the confidence interval. (TIFF) [file pone.0267444.s008.tiff]
