# Supplementary material for: Adaptive plasticity in activity modes and food web stability
Source: PLoS One. 2022 Apr 21;17(4):e0267444. doi: 10.1371/journal.pone.0267444 (PMC9022794; doi:10.1371/journal.pone.0267444)
Supplement: S1 File — (DOCX) [file pone.0267444.s009.docx]

**Mathematica codes for creating data of figures**

Note that a basic code of each figure is same.

(*code making data for Fig.1b*)

s = 40;(*species num*)

c = 0.3;(*connectance*)

gener = 5000;(*time*)

pa = 1;(*prop of adaptive species*)

rep = 500;(*replications*)

Mlis = {0.001`, 0.002`, 0.003`, 0.004`, 0.005`, 0.006`, 0.007`,

0.008`, 0.009, 0.01, 0.02`, 0.03`, 0.04`, 0.05`, 0.06`, 0.07`,

0.08`, 0.09, 0.1`, 0.2`, 0.3, 0.4`, 0.5`, 0.6`, 0.7`, 0.8`, 0.9,

1.`, 2, 3, 4, 5, 6, 7, 8, 9, 10};

(*list of adaptation speed*)

θlis = {0, 5, 10, 20};

(*list of accuracy*)

datalist = {};

Do[

data = {};

Do[

listlistpersit = {};

Do[

r1 = 5 RandomReal[{0, 1}, s];(*growth rate of active mode*)

r2 = 0.04 r1;(*growth rate of inactive mode*)

r = Join[r1, r2];(*growth rate*)

ε =

RandomReal[{1, 1}, 2 s];(*self regulation*)

m = M RandomReal[{0, 1}, s];(*adaptation speed*)

a1 = 0.1 RandomReal[{0, 1}, {s,

s}];(*consumption rate of active mode*)

a2 = 0.05 a1;(*consumption rate of inactive mode*)

g1 = RandomReal[{0.2, 0.2}, {s,

s}];(*conversion effec of active mode*)

g2 = 0.01 g1;(*conversion effec of inactive mode*)

xini = RandomReal[1, 2 s];(*initial pop size*)

(*making interaction network*)

NumPred = Round[0.5*s (s - 1)*c];

NumPrey = NumPred;

list =

Flatten[{Table[-1, {NumPred}],

Table[0, {0.5*s (s - 1) - NumPred}]}];

sample = RandomSample[Range[Length[list]], Length[list]];

sample = Table[list[[sample[[i]]]], {i, 1, Length[list]}];

plus = Reverse[Table[i, {i, 2, s - 1}]];

plus = Accumulate[plus] + Table[i, {i, 1, Length[plus]}];

Do[sample = Insert[sample, Table[0, {1 + i}], plus[[i]]], {i, 1,

Length[plus]}];

sample = PrependTo[sample, 0];

sample = Join[sample, Table[0, {s}]];

sample = Flatten[sample];

sample = Partition[sample, s];

pred1 = ReplacePart[sample, Position[sample, 1] -> 0];

pred2 = ReplacePart[sample, Position[sample, -1] -> 0];

pred3 =

ReplacePart[pred1, Position[pred1, -1] -> 1] + Transpose[pred2];

predpatch1 = -1*Transpose[pred1]*g1 + pred2*g1;

predpatch2 = -1*Transpose[pred1]*g2 + pred2*g2;

ichi = ReplacePart[sample, Position[sample, -1] -> 1];

ichipatch1 = ichi*a1 + Transpose[ichi*a1];

ichipatch2 = ichi*a2 + Transpose[ichi*a2];

intermatr = sample - Transpose[sample];

popsize1 = Table[Table[x1[i][n], {i, s}], {s}];

popsize2 = Table[Table[x2[i][n], {i, s}], {s}];

popsize = {}; AppendTo[popsize, Transpose[popsize1]];

AppendTo[popsize, Transpose[popsize2]];

popsize = Flatten[popsize, 1];

popsize = Flatten[Transpose[popsize]];

popsize = Flatten[AppendTo[popsize, popsize]];

popsize = Partition[popsize, 2 s];

interaction1 =

ichipatch1*intermatr*popsize1*(predpatch1 + pred3);

interaction2 =

ichipatch2*intermatr*popsize2*(predpatch2 + pred3);

diagonal =

r IdentityMatrix[2 s] -ε IdentityMatrix[2 s] popsize;

zero = Table[0, {i, s}];

Do[interaction1 = Insert[interaction1, zero, 2 i], {i, 1, s}];

interaction1 = Partition[Flatten[interaction1], 2 s];

Do[interaction2 = Insert[interaction2, zero, 2 i + 1], {i, 0, s}];

interaction2 = Partition[Flatten[interaction2], 2 s];

interaction = AppendTo[interaction1, interaction2];

interaction = Partition[Flatten[interaction], 2 s];

int = (interaction + diagonal);

(*making swithing function*)

Numadap = Round[s pa];

listadap = Flatten[{Table[1, {Numadap}], Table[0, {s - Numadap}]}];

sampleadap =

RandomSample[Range[Length[listadap]], Length[listadap]];

sampleadap =

Table[listadap[[sampleadap[[i]]]], {i, 1, Length[listadap]}];

mig1 =

Table[m[[i]]/(

1 + Exp[θ sampleadap[[

i]] (Total[int[[i]]] - Total[int[[i + s]]])]), {i, 1, s}];

mig2 = Table[

m[[i]] (1 -

1/(1 + Exp[θ sampleadap[[

i]] (Total[int[[i]]] - Total[int[[i + s]]])])), {i, 1,

s}];

popratio = (popsize1[[1]]/popsize2[[1]]);

popratio1 = (popratio^-1) mig2 IdentityMatrix[s];

popratio2 = (popratio) mig1 IdentityMatrix[s];

Do[popratio1 = Insert[popratio1, zero, 2 i + 1], {i, 0, s}];

popratio1 = Partition[Flatten[popratio1], 2 s];

Do[popratio2 = Insert[popratio2, zero, 2 i], {i, 1, s}];

popratio2 = Partition[Flatten[popratio2], 2 s];

migmatr = AppendTo[popratio1, popratio2];

migmatr = Partition[Flatten[migmatr], 2 s];

migdiagonal = Flatten[AppendTo[mig1, mig2]];

migdiagonal = -migdiagonal IdentityMatrix[2 s];

migmatr = migmatr + migdiagonal;

Interactionmatrix = (int + migmatr) popsize[[1]];

(*making diff equation*)

left1 = Table[x1[i]'[n], {i, s}];

left2 = Table[x2[i]'[n], {i, s}];

left = Join[left1, left2];

equation =

Table[left[[i]] == Total[Interactionmatrix[[i]]], {i, 1, 2 s}];

inipopsize1 = Table[x1[i][0], {i, s}];

inipopsize2 = Table[x2[i][0], {i, s}];

inipopsize = Join[inipopsize1, inipopsize2];

initial = Table[inipopsize[[i]] == xini[[i]], {i, 1, 2 s}];

equplusinitial = Join[equation, initial];

variable1 = Table[x1[i], {i, 1, s}];

variable2 = Table[x2[i], {i, 1, s}];

variable = Join[variable1, variable2];

kai =

NDSolve[equplusinitial, variable, {n, 0, gener},

MaxSteps -> Infinity, MaxStepSize -> Infinity];

listlast =

Flatten[Mean[

Table[Evaluate[popsize1[[1]] + popsize2[[1]] /. kai], {n,

gener - 500, gener}]]];

listpersist = {};

Do[If[listlast[[i]] > 10^-13,

AppendTo[listpersist, listlast[[i]]]], {i, 1, s}];

AppendTo[listlistpersit, Length[listpersist]/s], {rep}];

AppendTo[data, Count[listlistpersit, 1]/rep], {M, Mlis}];

AppendTo[datalist, data], {θ, θlis}]

datalist // N

(*code making data for Fig.2*)

s = 40; c = 0.3;

θ = 2;(*accuracy is varied*)

gener = 5000; M = 1; pa = 1;

rep = 500;

A2lis = {0, 0.001, 0.002, 0.003, 0.004, 0.005, 0.006, 0.007, 0.008,

0.009, 0.01, 0.02, 0.03, 0.04, 0.05, 0.06, 0.07, 0.08, 0.09, 0.1};

(*list of A2*)

datalist = {};

Do[

data = {};

Do[

listlistpersit = {};

Do[

r1 = 5 RandomReal[{0, 1}, s];

r2 = 0.04 r1;

r = Join[r1, r2];

ε = RandomReal[{1, 1}, 2 s];

m = M RandomReal[{0, 1}, s];

a1 = A1 RandomReal[{0, 1}, {s, s}];

a2 = A2 a1;

g1 = RandomReal[{0.2, 0.2}, {s, s}];

g2 = 0.01 g1;

xini = RandomReal[1, 2 s];

NumPred = Round[0.5*s (s - 1)*c];

NumPrey = NumPred;

list =

Flatten[{Table[-1, {NumPred}],

Table[0, {0.5*s (s - 1) - NumPred}]}];

sample = RandomSample[Range[Length[list]], Length[list]];

sample = Table[list[[sample[[i]]]], {i, 1, Length[list]}];

plus = Reverse[Table[i, {i, 2, s - 1}]];

plus = Accumulate[plus] + Table[i, {i, 1, Length[plus]}];

Do[sample = Insert[sample, Table[0, {1 + i}], plus[[i]]], {i, 1,

Length[plus]}];

sample = PrependTo[sample, 0];

sample = Join[sample, Table[0, {s}]];

sample = Flatten[sample];

sample = Partition[sample, s];

pred1 = ReplacePart[sample, Position[sample, 1] -> 0];

pred2 = ReplacePart[sample, Position[sample, -1] -> 0];

pred3 =

ReplacePart[pred1, Position[pred1, -1] -> 1] + Transpose[pred2];

predpatch1 = -1*Transpose[pred1]*g1 + pred2*g1;

predpatch2 = -1*Transpose[pred1]*g2 + pred2*g2;

ichi = ReplacePart[sample, Position[sample, -1] -> 1];

ichipatch1 = ichi*a1 + Transpose[ichi*a1];

ichipatch2 = ichi*a2 + Transpose[ichi*a2];

intermatr = sample - Transpose[sample];

popsize1 = Table[Table[x1[i][n], {i, s}], {s}];

popsize2 = Table[Table[x2[i][n], {i, s}], {s}];

popsize = {}; AppendTo[popsize, Transpose[popsize1]];

AppendTo[popsize, Transpose[popsize2]];

popsize = Flatten[popsize, 1];

popsize = Flatten[Transpose[popsize]];

popsize = Flatten[AppendTo[popsize, popsize]];

popsize = Partition[popsize, 2 s];

interaction1 =

ichipatch1*intermatr*popsize1*(predpatch1 + pred3);

interaction2 =

ichipatch2*intermatr*popsize2*(predpatch2 + pred3);

diagonal =

r IdentityMatrix[2 s] - ε IdentityMatrix[2 s] popsize;

zero = Table[0, {i, s}];

Do[interaction1 = Insert[interaction1, zero, 2 i], {i, 1, s}];

interaction1 = Partition[Flatten[interaction1], 2 s];

Do[interaction2 = Insert[interaction2, zero, 2 i + 1], {i, 0, s}];

interaction2 = Partition[Flatten[interaction2], 2 s];

interaction = AppendTo[interaction1, interaction2];

interaction = Partition[Flatten[interaction], 2 s];

int = (interaction + diagonal);

Numadap = Round[s pa];

listadap = Flatten[{Table[1, {Numadap}], Table[0, {s - Numadap}]}];

sampleadap =

RandomSample[Range[Length[listadap]], Length[listadap]];

sampleadap =

Table[listadap[[sampleadap[[i]]]], {i, 1, Length[listadap]}];

mig1 =

Table[m[[

i]]/(1 +

Exp[θ sampleadap[[

i]] (Total[int[[i]]] - Total[int[[i + s]]])]), {i, 1, s}];

mig2 = Table[

m[[i]] (1 -

1/(1 + Exp[θ sampleadap[[

i]] (Total[int[[i]]] - Total[int[[i + s]]])])), {i, 1,

s}];

popratio = (popsize1[[1]]/popsize2[[1]]);

popratio1 = (popratio^-1) mig2 IdentityMatrix[s];

popratio2 = (popratio) mig1 IdentityMatrix[s];

Do[popratio1 = Insert[popratio1, zero, 2 i + 1], {i, 0, s}];

popratio1 = Partition[Flatten[popratio1], 2 s];

Do[popratio2 = Insert[popratio2, zero, 2 i], {i, 1, s}];

popratio2 = Partition[Flatten[popratio2], 2 s];

migmatr = AppendTo[popratio1, popratio2];

migmatr = Partition[Flatten[migmatr], 2 s];

migdiagonal = Flatten[AppendTo[mig1, mig2]];

migdiagonal = -migdiagonal IdentityMatrix[2 s];

migmatr = migmatr + migdiagonal;

Interactionmatrix = (int + migmatr) popsize[[1]];

left1 = Table[x1[i]'[n], {i, s}];

left2 = Table[x2[i]'[n], {i, s}];

left = Join[left1, left2];

equation =

Table[left[[i]] == Total[Interactionmatrix[[i]]], {i, 1, 2 s}];

inipopsize1 = Table[x1[i][0], {i, s}];

inipopsize2 = Table[x2[i][0], {i, s}];

inipopsize = Join[inipopsize1, inipopsize2];

initial = Table[inipopsize[[i]] == xini[[i]], {i, 1, 2 s}];

equplusinitial = Join[equation, initial];

variable1 = Table[x1[i], {i, 1, s}];

variable2 = Table[x2[i], {i, 1, s}];

variable = Join[variable1, variable2];

kai =

NDSolve[equplusinitial, variable, {n, 0, gener},

MaxSteps -> Infinity, MaxStepSize -> Infinity];

listlast =

Flatten[Mean[

Table[Evaluate[popsize1[[1]] + popsize2[[1]] /. kai], {n,

gener - 500, gener}]]];

listpersist = {};

Do[If[listlast[[i]] > 10^-13,

AppendTo[listpersist, listlast[[i]]]], {i, 1, s}];

AppendTo[listlistpersit, Length[listpersist]/s], {rep}];

AppendTo[data, Count[listlistpersit, 1]/rep], {A1, 0.1, 1, 0.1}];

AppendTo[datalist, data], {A2, A2lis}]

datalist // N

(*code making data for Fig.3a*)

c = 0.3;

gener = 5000; pa = 1;

rep = 500; M = 1;

θlis = {0, 5, 10, 20};

slis = {5, 10, 15, 20, 25, 30, 35, 40, 45, 50};

datalist = {};

Do[

data = {};

Do[

listlistpersit = {};

Do[

r1 = 5 RandomReal[{0, 1}, s];

r2 = 0.04 r1;

r = Join[r1, r2];

ε = RandomReal[{1, 1}, 2 s];

m = M RandomReal[{0, 1}, s];

a1 = 0.1 RandomReal[{0, 1}, {s, s}];

a2 = 0.01 a1;

g1 = RandomReal[{0.2, 0.2}, {s, s}];

g2 = 0.01 g1;

xini = RandomReal[1, 2 s];

NumPred = Round[0.5*s (s - 1)*c];

NumPrey = NumPred;

list =

Flatten[{Table[-1, {NumPred}],

Table[0, {0.5*s (s - 1) - NumPred}]}];

sample = RandomSample[Range[Length[list]], Length[list]];

sample = Table[list[[sample[[i]]]], {i, 1, Length[list]}];

plus = Reverse[Table[i, {i, 2, s - 1}]];

plus = Accumulate[plus] + Table[i, {i, 1, Length[plus]}];

Do[sample = Insert[sample, Table[0, {1 + i}], plus[[i]]], {i, 1,

Length[plus]}];

sample = PrependTo[sample, 0];

sample = Join[sample, Table[0, {s}]];

sample = Flatten[sample];

sample = Partition[sample, s];

pred1 = ReplacePart[sample, Position[sample, 1] -> 0];

pred2 = ReplacePart[sample, Position[sample, -1] -> 0];

pred3 =

ReplacePart[pred1, Position[pred1, -1] -> 1] + Transpose[pred2];

predpatch1 = -1*Transpose[pred1]*g1 + pred2*g1;

predpatch2 = -1*Transpose[pred1]*g2 + pred2*g2;

ichi = ReplacePart[sample, Position[sample, -1] -> 1];

ichipatch1 = ichi*a1 + Transpose[ichi*a1];

ichipatch2 = ichi*a2 + Transpose[ichi*a2];

intermatr = sample - Transpose[sample];

popsize1 = Table[Table[x1[i][n], {i, s}], {s}];

popsize2 = Table[Table[x2[i][n], {i, s}], {s}];

popsize = {}; AppendTo[popsize, Transpose[popsize1]];

AppendTo[popsize, Transpose[popsize2]];

popsize = Flatten[popsize, 1];

popsize = Flatten[Transpose[popsize]];

popsize = Flatten[AppendTo[popsize, popsize]];

popsize = Partition[popsize, 2 s];

interaction1 =

ichipatch1*intermatr*popsize1*(predpatch1 + pred3);

interaction2 =

ichipatch2*intermatr*popsize2*(predpatch2 + pred3);

diagonal =

r IdentityMatrix[2 s] - ε IdentityMatrix[2 s] popsize;

zero = Table[0, {i, s}];

Do[interaction1 = Insert[interaction1, zero, 2 i], {i, 1, s}];

interaction1 = Partition[Flatten[interaction1], 2 s];

Do[interaction2 = Insert[interaction2, zero, 2 i + 1], {i, 0, s}];

interaction2 = Partition[Flatten[interaction2], 2 s];

interaction = AppendTo[interaction1, interaction2];

interaction = Partition[Flatten[interaction], 2 s];

int = (interaction + diagonal);

Numadap = Round[s pa];

listadap = Flatten[{Table[1, {Numadap}], Table[0, {s - Numadap}]}];

sampleadap =

RandomSample[Range[Length[listadap]], Length[listadap]];

sampleadap =

Table[listadap[[sampleadap[[i]]]], {i, 1, Length[listadap]}];

mig1 =

Table[m[[i]]/(

1 + Exp[θ sampleadap[[

i]] (Total[int[[i]]] - Total[int[[i + s]]])]), {i, 1, s}];

mig2 = Table[

m[[i]] (1 -

1/(1 + Exp[θ sampleadap[[

i]] (Total[int[[i]]] - Total[int[[i + s]]])])), {i, 1,

s}];

popratio = (popsize1[[1]]/popsize2[[1]]);

popratio1 = (popratio^-1) mig2 IdentityMatrix[s];

popratio2 = (popratio) mig1 IdentityMatrix[s];

Do[popratio1 = Insert[popratio1, zero, 2 i + 1], {i, 0, s}];

popratio1 = Partition[Flatten[popratio1], 2 s];

Do[popratio2 = Insert[popratio2, zero, 2 i], {i, 1, s}];

popratio2 = Partition[Flatten[popratio2], 2 s];

migmatr = AppendTo[popratio1, popratio2];

migmatr = Partition[Flatten[migmatr], 2 s];

migdiagonal = Flatten[AppendTo[mig1, mig2]];

migdiagonal = -migdiagonal IdentityMatrix[2 s];

migmatr = migmatr + migdiagonal;

Interactionmatrix = (int + migmatr) popsize[[1]];

left1 = Table[x1[i]'[n], {i, s}];

left2 = Table[x2[i]'[n], {i, s}];

left = Join[left1, left2];

equation =

Table[left[[i]] == Total[Interactionmatrix[[i]]], {i, 1, 2 s}];

inipopsize1 = Table[x1[i][0], {i, s}];

inipopsize2 = Table[x2[i][0], {i, s}];

inipopsize = Join[inipopsize1, inipopsize2];

initial = Table[inipopsize[[i]] == xini[[i]], {i, 1, 2 s}];

equplusinitial = Join[equation, initial];

variable1 = Table[x1[i], {i, 1, s}];

variable2 = Table[x2[i], {i, 1, s}];

variable = Join[variable1, variable2];

kai =

NDSolve[equplusinitial, variable, {n, 0, gener},

MaxSteps -> Infinity, MaxStepSize -> Infinity];

listlast =

Flatten[Mean[

Table[Evaluate[popsize1[[1]] + popsize2[[1]] /. kai], {n,

gener - 500, gener}]]];

listpersist = {};

Do[If[listlast[[i]] > 10^-13,

AppendTo[listpersist, listlast[[i]]]], {i, 1, s}];

AppendTo[listlistpersit, Length[listpersist]/s], {rep}];

AppendTo[data, Count[listlistpersit, 1]/rep], {s, slis}];

AppendTo[datalist, data], {θ, θlis}] // Timing

datalist // N

(*code making data for Fig.3b*)

s = 40;

gener = 2000; pa = 1;

rep = 500; M = 1;

θlis = {0, 5, 10, 20};

datalist = {};

Do[

data = {};

Do[

listlistpersit = {};

Do[

r1 = 5 RandomReal[{0, 1}, s];

r2 = 0.04 r1;

r = Join[r1, r2];

ε= RandomReal[{1, 1}, 2 s];

m = M RandomReal[{0, 1}, s];

a1 = 0.1 RandomReal[{0, 1}, {s, s}];

a2 = 0.01 a1;

g1 = RandomReal[{0.2, 0.2}, {s, s}];

g2 = 0.01 g1;

xini = RandomReal[1, 2 s];

NumPred = Round[0.5*s (s - 1)*c];

NumPrey = NumPred;

list =

Flatten[{Table[-1, {NumPred}],

Table[0, {0.5*s (s - 1) - NumPred}]}];

sample = RandomSample[Range[Length[list]], Length[list]];

sample = Table[list[[sample[[i]]]], {i, 1, Length[list]}];

plus = Reverse[Table[i, {i, 2, s - 1}]];

plus = Accumulate[plus] + Table[i, {i, 1, Length[plus]}];

Do[sample = Insert[sample, Table[0, {1 + i}], plus[[i]]], {i, 1,

Length[plus]}];

sample = PrependTo[sample, 0];

sample = Join[sample, Table[0, {s}]];

sample = Flatten[sample];

sample = Partition[sample, s];

pred1 = ReplacePart[sample, Position[sample, 1] -> 0];

pred2 = ReplacePart[sample, Position[sample, -1] -> 0];

pred3 =

ReplacePart[pred1, Position[pred1, -1] -> 1] + Transpose[pred2];

predpatch1 = -1*Transpose[pred1]*g1 + pred2*g1;

predpatch2 = -1*Transpose[pred1]*g2 + pred2*g2;

ichi = ReplacePart[sample, Position[sample, -1] -> 1];

ichipatch1 = ichi*a1 + Transpose[ichi*a1];

ichipatch2 = ichi*a2 + Transpose[ichi*a2];

intermatr = sample - Transpose[sample];

popsize1 = Table[Table[x1[i][n], {i, s}], {s}];

popsize2 = Table[Table[x2[i][n], {i, s}], {s}];

popsize = {}; AppendTo[popsize, Transpose[popsize1]];

AppendTo[popsize, Transpose[popsize2]];

popsize = Flatten[popsize, 1];

popsize = Flatten[Transpose[popsize]];

popsize = Flatten[AppendTo[popsize, popsize]];

popsize = Partition[popsize, 2 s];

interaction1 =

ichipatch1*intermatr*popsize1*(predpatch1 + pred3);

interaction2 =

ichipatch2*intermatr*popsize2*(predpatch2 + pred3);

diagonal =

r IdentityMatrix[2 s] - ε IdentityMatrix[2 s] popsize;

zero = Table[0, {i, s}];

Do[interaction1 = Insert[interaction1, zero, 2 i], {i, 1, s}];

interaction1 = Partition[Flatten[interaction1], 2 s];

Do[interaction2 = Insert[interaction2, zero, 2 i + 1], {i, 0, s}];

interaction2 = Partition[Flatten[interaction2], 2 s];

interaction = AppendTo[interaction1, interaction2];

interaction = Partition[Flatten[interaction], 2 s];

int = (interaction + diagonal);

Numadap = Round[s pa];

listadap = Flatten[{Table[1, {Numadap}], Table[0, {s - Numadap}]}];

sampleadap =

RandomSample[Range[Length[listadap]], Length[listadap]];

sampleadap =

Table[listadap[[sampleadap[[i]]]], {i, 1, Length[listadap]}];

mig1 =

Table[m[[i]]/(

1 + Exp[θ sampleadap[[

i]] (Total[int[[i]]] - Total[int[[i + s]]])]), {i, 1, s}];

mig2 = Table[

m[[i]] (1 -

1/(1 + Exp[θ sampleadap[[

i]] (Total[int[[i]]] - Total[int[[i + s]]])])), {i, 1,

s}];

popratio = (popsize1[[1]]/popsize2[[1]]);

popratio1 = (popratio^-1) mig2 IdentityMatrix[s];

popratio2 = (popratio) mig1 IdentityMatrix[s];

Do[popratio1 = Insert[popratio1, zero, 2 i + 1], {i, 0, s}];

popratio1 = Partition[Flatten[popratio1], 2 s];

Do[popratio2 = Insert[popratio2, zero, 2 i], {i, 1, s}];

popratio2 = Partition[Flatten[popratio2], 2 s];

migmatr = AppendTo[popratio1, popratio2];

migmatr = Partition[Flatten[migmatr], 2 s];

migdiagonal = Flatten[AppendTo[mig1, mig2]];

migdiagonal = -migdiagonal IdentityMatrix[2 s];

migmatr = migmatr + migdiagonal;

Interactionmatrix = (int + migmatr) popsize[[1]];

left1 = Table[x1[i]'[n], {i, s}];

left2 = Table[x2[i]'[n], {i, s}];

left = Join[left1, left2];

equation =

Table[left[[i]] == Total[Interactionmatrix[[i]]], {i, 1, 2 s}];

inipopsize1 = Table[x1[i][0], {i, s}];

inipopsize2 = Table[x2[i][0], {i, s}];

inipopsize = Join[inipopsize1, inipopsize2];

initial = Table[inipopsize[[i]] == xini[[i]], {i, 1, 2 s}];

equplusinitial = Join[equation, initial];

variable1 = Table[x1[i], {i, 1, s}];

variable2 = Table[x2[i], {i, 1, s}];

variable = Join[variable1, variable2];

kai =

NDSolve[equplusinitial, variable, {n, 0, gener},

MaxSteps -> Infinity, MaxStepSize -> Infinity];

listlast =

Flatten[Mean[

Table[Evaluate[popsize1[[1]] + popsize2[[1]] /. kai], {n,

gener - 500, gener}]]];

listpersist = {};

Do[If[listlast[[i]] > 10^-13,

AppendTo[listpersist, listlast[[i]]]], {i, 1, s}];

AppendTo[listlistpersit, Length[listpersist]/s], {rep}];

AppendTo[data, Count[listlistpersit, 1]/rep], {c, 0.1, 1, 0.1}];

AppendTo[datalist, data], {θ, θlis}] // Timing

datalist // N

(*code making data for Fig.4*)

s = 40; c = 0.3;

gener = 5000; pa = 1;

A1 = 0.01; (*A12 in text*)

A2 = 0.005;(*A21 in text*)

A3 = 0.001;(*A22 in text*)

rep = 500;

Mlis = {0.001`, 0.002`, 0.003`, 0.004`, 0.005`, 0.006`, 0.007`,

0.008`, 0.009, 0.01, 0.02`, 0.03`, 0.04`, 0.05`, 0.06`, 0.07`,

0.08`, 0.09, 0.1`, 0.2`, 0.3, 0.4`, 0.5`, 0.6`, 0.7`, 0.8`, 0.9,

1.`, 2, 3, 4, 5, 6, 7, 8, 9, 10};(*list of adaptation speed*)

θlis = {0, 2, 5, 10, 20};

datalist = {};

Do[

data = {};

Do[

listlistpersist = {};

Do[

r1 = 5 RandomReal[{0, 1}, s];

r2 = 0.04 r1;

r = Join[r1, r2];

ε1 = RandomReal[{1, 1}, s];

ε2 = 0.5 ε1;

ε = Join[ε1, ε2];

m = M RandomReal[{0, 1}, s];

a1 = 0.1 RandomReal[{0, 1}, {s, s}];

a2 = A1 a1;

a3 = A2 a1;

a4 = A3 a1;

g1 = RandomReal[{0.2, 0.2}, {s, s}];

g2 = 0.01 g1;

xini = RandomReal[1, 2 s];

NumPred = Round[0.5*s (s - 1)*c];

NumPrey = NumPred;

list =

Flatten[{Table[-1, {NumPred}],

Table[0, {0.5*s (s - 1) - NumPred}]}];

sample = RandomSample[Range[Length[list]], Length[list]];

sample = Table[list[[sample[[i]]]], {i, 1, Length[list]}];

plus = Reverse[Table[i, {i, 2, s - 1}]];

plus = Accumulate[plus] + Table[i, {i, 1, Length[plus]}];

Do[sample = Insert[sample, Table[0, {1 + i}], plus[[i]]], {i, 1,

Length[plus]}];

sample = PrependTo[sample, 0];

sample = Join[sample, Table[0, {s}]];

sample = Flatten[sample];

sample = Partition[sample, s];

pred1 = ReplacePart[sample, Position[sample, 1] -> 0];

pred2 = ReplacePart[sample, Position[sample, -1] -> 0];

pred3 = ReplacePart[pred1, Position[pred1, -1] -> 1] +

Transpose[pred2];

pred11 = -1*Transpose[pred1]*g1 + pred2*g1;

pred12 = -1*Transpose[pred1]*g1 + pred2*g1;

pred21 = -1*Transpose[pred1]*g2 + pred2*g2;

pred22 = -1*Transpose[pred1]*g2 + pred2*g2;

ichi = ReplacePart[sample, Position[sample, -1] -> 1];

ichi11 = ichi*a1 + Transpose[ichi*a1];

ichi12 = Transpose[ichi*a2] + ichi*a3;

ichi21 = Transpose[ichi*a3] + ichi*a2;

ichi22 = ichi*a4 + Transpose[ichi*a4];

intermatr = sample - Transpose[sample];

popsize1 = Table[Table[x1[i][n], {i, s}], {s}];

popsize2 = Table[Table[x2[i][n], {i, s}], {s}];

popsize = {}; AppendTo[popsize, Transpose[popsize1]];

AppendTo[popsize, Transpose[popsize2]];

popsize = Flatten[popsize, 1];

popsize = Flatten[Transpose[popsize]];

popsize = Flatten[AppendTo[popsize, popsize]];

popsize = Partition[popsize, 2 s];

interaction1 = ichi11*intermatr*popsize1*(pred11 + pred3);

interaction2 = ichi12*intermatr*popsize2*(pred12 + pred3);

interaction3 = ichi21*intermatr*popsize1*(pred21 + pred3);

interaction4 = ichi22*intermatr*popsize2*(pred22 + pred3);

zero = Table[0, {i, s}];

Do[interaction1 = Insert[interaction1, zero, 2 i], {i, 1, s}];

interaction1 = Partition[Flatten[interaction1], 2 s];

Do[interaction2 = Insert[interaction2, zero, 2 i + 1], {i, 0,

s}];

interaction2 = Partition[Flatten[interaction2], 2 s];

Do[interaction3 = Insert[interaction3, zero, 2 i], {i, 1, s}];

interaction3 = Partition[Flatten[interaction3], 2 s];

Do[interaction4 = Insert[interaction4, zero, 2 i + 1], {i, 0,

s}];

interaction4 = Partition[Flatten[interaction4], 2 s];

interactionactive = interaction1 + interaction2;

interactioninactive = interaction3 + interaction4;

interaction = AppendTo[interactionactive, interactioninactive];

interaction = Partition[Flatten[interaction], 2 s];

diagonal =

r IdentityMatrix[2 s] - ε IdentityMatrix[2 s] popsize;

int = (interaction + diagonal);

Numadap = Round[s pa];

listadap = Flatten[{Table[1, {Numadap}], Table[0, {s - Numadap}]}];

sampleadap =

RandomSample[Range[Length[listadap]], Length[listadap]];

sampleadap =

Table[listadap[[sampleadap[[i]]]], {i, 1, Length[listadap]}];

mig1 =

Table[m[[i]]/(

1 + Exp[θ sampleadap[[

i]] (Total[int[[i]]] - Total[int[[i + s]]])]), {i, 1, s}];

mig2 = Table[

m[[i]] (1 - 1/(

1 + Exp[θ sampleadap[[

i]] (Total[int[[i]]] - Total[int[[i + s]]])])), {i, 1,

s}];

popratio = (popsize1[[1]]/popsize2[[1]]);

popratio1 = (popratio^-1) mig2 IdentityMatrix[s];

popratio2 = (popratio) mig1 IdentityMatrix[s];

Do[popratio1 = Insert[popratio1, zero, 2 i + 1], {i, 0, s}];

popratio1 = Partition[Flatten[popratio1], 2 s];

Do[popratio2 = Insert[popratio2, zero, 2 i], {i, 1, s}];

popratio2 = Partition[Flatten[popratio2], 2 s];

migmatr = AppendTo[popratio1, popratio2];

migmatr = Partition[Flatten[migmatr], 2 s];

migdiagonal = Flatten[AppendTo[mig1, mig2]];

migdiagonal = -migdiagonal IdentityMatrix[2 s];

migmatr = migmatr + migdiagonal;

Interactionmatrix = (int + migmatr) popsize[[1]];

left1 = Table[x1[i]'[n], {i, s}];

left2 = Table[x2[i]'[n], {i, s}];

left = Join[left1, left2];

equation =

Table[left[[i]] == Total[Interactionmatrix[[i]]], {i, 1, 2 s}];

inipopsize1 = Table[x1[i][0], {i, s}];

inipopsize2 = Table[x2[i][0], {i, s}];

inipopsize = Join[inipopsize1, inipopsize2];

initial = Table[inipopsize[[i]] == xini[[i]], {i, 1, 2 s}];

equplusinitial = Join[equation, initial];

variable1 = Table[x1[i], {i, 1, s}];

variable2 = Table[x2[i], {i, 1, s}];

variable = Join[variable1, variable2];

kai =

NDSolve[equplusinitial, variable, {n, 0, gener},

MaxSteps -> Infinity, MaxStepSize -> Infinity];

listlast =

Flatten[Mean[

Table[Evaluate[popsize1[[1]] + popsize2[[1]] /. kai], {n,

gener - 500, gener}]]];

meanswitch =

Mean[Flatten[

Mean[Table[

Evaluate[-(mig2/m - 1) /. kai], {n, gener - 500,

gener}]]]];(*swithing probability at equlibrium*)

listpersist = {};

Do[If[listlast[[i]] > 10^-13,

AppendTo[listpersist, listlast[[i]]]], {i, 1, s}];

AppendTo[

listlistpersist, {Length[listpersist]/s, meanswitch}], {rep}];

AppendTo[

data, {Count[Transpose[listlistpersist][[1]], 1]/rep,

Mean[Transpose[listlistpersist][[2]]],

StandardDeviation[Transpose[listlistpersist][[2]]]}], {M, Mlis}];

AppendTo[datalist, data], {θ, θlis}] // Timing

datalist//N
